# Bioinformatics analysis reveals biophysical and evolutionary insights into the 3-nitrotyrosine post-translational modification in the human proteome

**DOI:** 10.1098/rsob.120148

**Published:** 2013-02

**Authors:** John Y. Ng, Lies Boelen, Jason W. H. Wong

**Affiliations:** 1 Lowy Cancer Research Centre, Prince of Wales Clinical School, University of New South Wales, Sydney 2052, Australia; 2 School of Medical Sciences, University of New South Wales, Sydney 2052, Australia

**Keywords:** 3-nitrotyrosine, bioinformatics, secondary structure, post-translational modification conservation, phosphotyrosine

## Abstract

Protein 3-nitrotyrosine is a post-translational modification that commonly arises from the nitration of tyrosine residues. This modification has been detected under a wide range of pathological conditions and has been shown to alter protein function. Whether 3-nitrotyrosine is important in normal cellular processes or is likely to affect specific biological pathways remains unclear. Using GPS-YNO2, a recently described 3-nitrotyrosine prediction algorithm, a set of predictions for nitrated residues in the human proteome was generated. In total, 9.27 per cent of the proteome was predicted to be nitratable (27 922/301 091). By matching the predictions against a set of curated and experimentally validated 3-nitrotyrosine sites in human proteins, it was found that GPS-YNO2 is able to predict 73.1 per cent (404/553) of these sites. Furthermore, of these sites, 42 have been shown to be nitrated endogenously, with 85.7 per cent (36/42) of these predicted to be nitrated. This demonstrates the feasibility of using the predicted dataset for a whole proteome analysis. A comprehensive bioinformatics analysis was subsequently performed on predicted and all experimentally validated nitrated tyrosine. This found mild but specific biophysical constraints that affect the susceptibility of tyrosine to nitration, and these may play a role in increasing the likelihood of 3-nitrotyrosine to affect processes, including phosphorylation and DNA binding. Furthermore, examining the evolutionary conservation of predicted 3-nitrotyrosine showed that, relative to non-nitrated tyrosine residues, 3-nitrotyrosine residues are generally less conserved. This suggests that, at least in the majority of cases, 3-nitrotyrosine is likely to have a deleterious effect on protein function and less likely to be important in normal cellular function.

## 2. Introduction

Protein 3-nitrotyrosine is a post-translational modification (PTM) of the amino acid tyrosine, with the covalent substitution of a nitrite group positioned in the 3-position in the aromatic phenol of tyrosine [1]. The modification is mediated by reactive nitrogen species and can be formed under a number of physiological conditions [2–4]. One of the most well-known pathways through which tyrosine is nitrated is from the reactive nitrating intermediate peroxynitrite (ONOO^−^). Peroxynitrite is formed *in vivo* via the fast reaction between superoxide (O_2_^−^) and nitric oxide (NO^−^) produced by NADPH oxidase and the enzyme nitric oxide synthase, respectively [5,6]. As such, the 3-nitrotyrosine has been found to be elevated during inflammatory processes where immuno-inflammatory cells and nitric oxide levels are elevated [7,8]. As inflammation has been linked to a wide range of human pathological conditions, 3-nitrotyrosine has now also been observed in a large number of diseases, including lung cancer, cardiovascular disease, asthma and Alzheimer's disease [9–14].

Although many studies have identified the presence of 3-nitrotyrosine, knowledge of how this PTM generally affects protein function remains limited [15–17]. The nitro functional group represents a bulky neutral moiety that is likely to have an influence in the normal biology of tyrosine. Indeed, crystal structure analyses of manganese superoxide dismutase show that the inhibition of catalytic activity is due to the steric effect of 3-nitrotyrosine in impeding substrate access and binding. Furthermore, the close proximity of 3-nitrotyrosine to a glutamine in the active site alters the hydrogen bond network that supports proton transfer in catalysis [18]. In a separate study, the protein thioredoxin-1 displayed inactivation by 3-nitrotyrosine, blocking the protein's natural anti-apoptotic and cardio-protective effects [19,20]. Furthermore, it has been proposed that the nitration of tyrosine may directly prevent phosphorylation of the same residue, thereby preventing important phosphotyrosine-mediated signalling cascades [21–24]. Conversely, at the cellular level, tyrosine nitration has been shown to increase the overall level of phosphotyrosine in the proteome [23,25–28]. Collectively, current evidence suggests that there is potential biological significance of the formation of 3-nitrotyrosine in causing abnormal cellular function.

The analysis of 3-nitrotyrosine in biological samples has presented challenges [29]. In part, this is probably owing to the low abundance of the PTM, limiting the ability to pinpoint the specific sites of tyrosine nitration. To date, the majority of studies have been performed using immunoaffinity-based techniques to identify proteins that are tyrosine-nitrated [30,31]. A limitation of these studies is that they are generally unable to pinpoint the specific tyrosine residues that are affected. Furthermore, the possibility of the antibody binding to non-specific epitopes is difficult to exclude in experiments, thus this may result in false positive detection. In recent years, development of derivatization strategies coupled with modern mass spectrometry analysis has allowed substantial progress to be made in terms of the identification of specific proteins and sites of tyrosine nitration under both *in vitro* and *in vivo* conditions [32–34]. By reducing and therefore functionalizing the 3-nitrotyrosine into a 3-aminotyrosine and pre-blocking other primary amines, Zhang *et al*. [35] were able to specifically biotinylate, enrich and identify 150 unique 3-nitrotyrosine-containing peptides corresponding to 102 proteins from an *in vitro* nitrated rat brain extract. More recently, Ghesquiere *et al*. [36] applied diagonal chromatography by taking advantage of a hydrophilic shift upon the reduction of 3-nitrotyrosine into 3-aminotyrosine to identity 3-nitrotyrosines. In that study, 335 3-nitrotyrosine-containing peptides corresponding to 267 proteins in an *in vitro* nitrated Jurkat cell extract and six 3-nitrotyrosine-containing peptides from four mouse proteins extracted from mouse serum were successfully identified and enriched [36].

Early studies suggested that there is either no consensus motif around nitrated tyrosine residues [37,38] or, even if there is, it is probably loosely defined [39]. Nevertheless, with a growing body of experimentally verified nitration sites, a computational model (GPS-YNO2) was developed for predicting tyrosine residues that are susceptible to nitration based on the biochemical properties of amino acids adjacent to tyrosine [40]. Using cross-validation, the model was shown to predict 3-nitrotyrosine with an accuracy of over 76 per cent and a specificity of 80.18 per cent.

The lack of experimentally verified susceptible sites has made it difficult to examine global biophysical and evolutionary trends of 3-nitrotyrosine. The ability to predict 3-nitrotyrosine provides an opportunity to examine the impact of this PTM at the proteome level*.* Using the reference human proteome, a comprehensive computational analysis of tyrosine residues predicted to be susceptible to nitration was performed. The prediction of 3-nitrotyrosine residues was first validated against sites that have been previously experimentally identified in human proteins. Subsequently, some biophysical and biochemical properties of predicted and experimentally validated 3-nitrotyrosine residues were examined. Furthermore, the overlap of tyrosine residues that are susceptible to both phosphorylation and nitration was analysed. Finally, by tracking the conservation of tyrosine across mammalian orthologous proteins, whether nitrated tyrosine residues are under selective pressure in natural protein evolution was examined.

## 3. Material and methods

### 3.1. Prediction of 3-nitrotyrosine in the human proteome

Canonical protein sequences from all curated human proteins were obtained in FASTA format from the UniProt/Swiss-Prot database (release 2012_04). To predict 3-nitrotyrosine in all human proteins, the local version of GPS-YNO2 (v. 1.0) was used. The batch predictor tool was used to import and analyse all human proteins. All threshold levels (high, medium and low) were used initially to generate a complete list of predicted 3-nitrotyrosine sites (see electronic supplementary material, table S1 for list of counts). Since the high threshold still resulted in a relatively large number of 3-nitrotyrosine sites, for all ensuing analysis, only these sites were used (27 922 tyrosine residues). The list of predicted sites was merged with UniProt sequence annotations and exported for further analysis. A full table summarizing the annotations of all tyrosine residues in human UniProt proteins can be found in the electronic supplementary material, table S2.

### 3.2. Curation of experimentally identified 3-nitrotyrosine residues in human proteins

Human protein nitration sites retrieved from supplementary table S1 of Liu *et al*. [40] and from literature published since 2011 retrieved from PubMed using the search term ‘tyrosine nitration’ were reviewed manually. In total, 553 tyrosine sites have experimental evidence for nitration. The proteins and sites were determined to be endogenously nitrated *in vivo* only if they had been identified from untreated human samples using mass spectrometry. The *in vivo* sites were then matched with the whole predicted human tyrosine proteome dataset using the UniProt/Swiss-Prot accession and labelled accordingly in electronic supplementary material, table S2. This resulted in a final set of 42 *in vivo* validated 3-nitrotyrosine sites in the human proteome.

### 3.3. Gene-annotation enrichment analysis of tyrosine-nitrated proteins

All human proteins that had one or more predicted nitrated sites were considered to be nitrated (12 338), while those without any predicted nitration sites were considered not to be nitrated (7407). Gene annotation enrichment analysis was performed using DAVID [41], with nitrated proteins as the gene set and the non-nitrated proteins as background. Enriched biological themes were determined using the UniProt keyword annotations. As a further control for enrichment, random lists of 12 338 proteins were generated to validate that features reported to be enriched in nitrated proteins are indeed true positives.

### 3.4. Structural analysis of tyrosine susceptible to nitration

As of April 2012, there were 15 466 X-ray crystal structures of human proteins in the Protein Data Bank (PDB) which map to 3500 proteins from UniProt/Swiss-Prot database. Of these, 2399 nitrated proteins contain 5815 predicted 3-nitrotyrosine sites, while 35 nitrated proteins contain 81 experimentally validated 3-nitrotyrosine sites. To determine the solvent accessibility and the secondary structure of tyrosine residues, all X-ray structures were downloaded in PDB format and analysed using DSSP [42]. The solvent accessibility values were used as generated by DSSP. The secondary structural information was further grouped into helix (H, G, I), strand (B, E), turn (T, S) and loop/random (blank), where the corresponding DSSP output code is listed in the brackets.

### 3.5. Two Sample Logo analysis

To examine the amino acid composition adjacent to tyrosine residues, Two Sample Logo [43] was used. The Two Sample Logo tool generates a sequence logo that shows amino acids that are statistically significantly enriched or depleted across sequences generated under two conditions. Sequences ±10 residues of all tyrosine residues were aligned based on the tyrosine residue of interest. Where an N- or C-terminus falls within the ±10 residues, the remaining residues were padded with gaps. To generate the Two Sample Logos, the sequences centred around potentially nitrated tyrosine were compared with sequences centred around non-nitratable tyrosine residues. The *t*-test was selected to evaluate statistical significance and colouring of residues was based on α-helix propensity as described by Pace & Scholtz [44] or based on charge.

### 3.6. Overlap with phosphotyrosine residues

Phosphotyrosine annotations were obtained from PhosphoSitePlus [45] (12 268). To determine whether predicted and experimentally validated 3-nitrotyrosine are more likely to overlap with phosphorylated tyrosine residues than by chance, an equal number of all tyrosine residues were randomly selected and counted for overlap with phosphotyrosine residues. The bootstrapping process was repeated 500 times to assess statistical significance.

### 3.7. Conservation of nitrated tyrosine residues

To determine the conservation of tyrosine through protein evolution, orthologous proteins from 10 mammalian species (*Homo sapiens*, *Pan troglodytes*, *Mus musculus*, *Rattus norvegicus*, *Bos taurus*, *Sus scrofa*, *Equus caballus*, *Canis familiaris*, *Monodelphis domestica* and *Ornithorhynchus anatinus*) were compared. Orthologous protein clusters and sequences were obtained from OrthoDB (v. 5) [46]. Only orthologous clusters where the human protein can be mapped to a UniProt accession and where all 10 species are present were used for analysis of conservation (6394). The protein sequences for each species from each cluster were then retrieved and aligned with ClustalW2 [47] using default parameters. To determine the conservation, a tyrosine is considered non-conserved if it was substituted by another amino acid or by a gap of fewer than five residues in length. The choice of gap size is based on the observation that gaps of moderate size are often the result of a missing protein domain or the truncation of a protein and are likely to have arisen from a more significant change in protein function than can be attributed to the loss or gain of a tyrosine residue. To quantify the conservation of each tyrosine, normalized Shannon's entropy, *C*, [48] was used,2.1
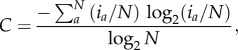
where *N* is the number of types of residues found and *i_a_* is the number of residues of type *a*. A higher value (upper limit of 1) represents a site with a high number of substitutions, while a fully conserved site will have a value of zero. A tyrosine was considered to be conserved if *C* was zero and not conserved if *C* was greater than zero. The conservation status of all tyrosine residues with orthologous proteins across the 10 mammalian species is summarized in electronic supplementary material, table S3.

## 4. Results

### 4.1. GPS-YNO2 predicts *in vivo* 3-nitrotyrosine residues with good accuracy

To ensure that the 3-nitrotyrosine predictions by GPS-YNO2 are representative of the true state of tyrosine nitration *in vivo*, experimentally identified *in vivo* nitrated residues from the literature were manually curated. This resulted in the identification of 42 sites, of which 36 (85.7%) were also predicted to be nitrated by GPS-YNO2 (see electronic supplementary material, table S2 for a list of these sites).

### 4.2. Frequency of 3-nitrotyrosine correlates with total tyrosine residues in proteins

While the formation of many PTMs is catalysed by specific enzymes, and in particular both the formation and reversal of almost all regulatory modifications are catalysed by specific enzymes, to date, none has been identified for tyrosine nitration. To investigate the biophysical and evolutionary trends of tyrosine nitration on the human proteome, tyrosine residues were annotated as nitrated or non-nitrated based on the prediction of GPS-YNO2 [40].

**Figure 1.**
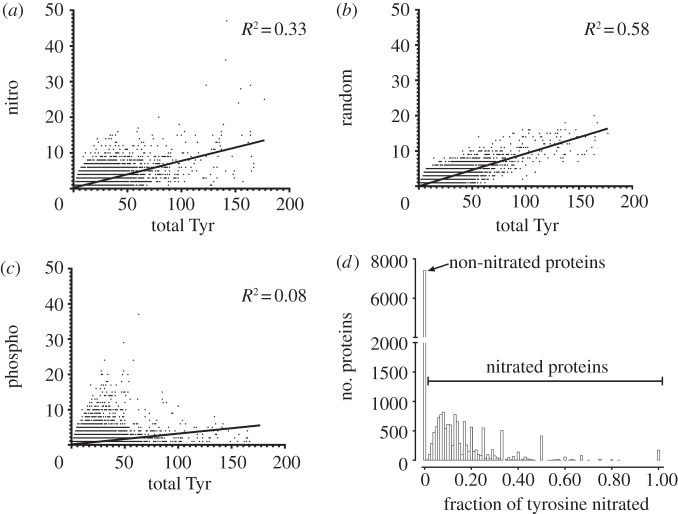
Distribution of (*a*) nitrated, (*b*) random (representative from 10 trials) and (*c*) phosphorylated tyrosine residues versus total tyrosine residues in UniProt/Swiss-Prot proteins. The distribution of the fraction of tyrosine residues that are nitrated is also shown as a histogram (*d*) where, for this study, any protein with no nitration sites is considered to be non-nitrated while proteins with any tyrosine residue nitrated is considered to be nitrated.

To determine whether the occurrence of 3-nitrotyrosine correlates with the frequency of occurrence of tyrosine in proteins, the number of 3-nitrotyrosine was correlated with the total number of tyrosine residues in each protein (figure 1*a*). As control, an equal number of tyrosine residues were randomly selected across the proteome and these random tyrosine residues were also correlated with total tyrosine in their respective protein (figure 1*b*). 3-nitrotyrosine residues show moderate correlation with total tyrosine (*R*^2^ = 0.334) when compared with the correlation between randomly distributed tyrosine and total tyrosine (*R*^2^ = 0.576). The difference between the distribution of 3-nitrotyrosine was further compared with randomly selected tyrosine residues and was found to be significantly different (*p* < 0.0001, paired two-tailed *t*-test). In contrast, the distribution of different sets of randomly generated tyrosine residues was never significant over 10 trials (*p* > 0.2, paired two-tailed *t*-test). This indicates that while the numbers of predicted 3-nitrotyrosine residues generally increase with numbers of tyrosine residues in a protein, it is nevertheless not completely random.

To determine how the distribution of 3-nitrotyrosine compare with those of PTMs with known enzymatic regulation, the correlation of phosphotyrosine and total tyrosine residues was also determined (figure 1*c*). This correlation is significantly lower compared with 3-nitrotyrosines (*R*^2^ = 0.076; *p* < 0.0001, paired two-tailed *t*-test).

### 4.3. Proteins susceptible to tyrosine nitration are enriched for specific biological features

All proteins were then separated into two categories (nitrated or non-nitrated) based on whether there is any nitrated tyrosine at all in a particular protein (figure 1*d*). The nitrated proteins were evaluated for the enrichment of biological features using DAVID (table 1). The most significantly enriched terms are ‘phosphoprotein’ and ‘coiled coil’.

**Table 1.** Top 10 enriched UniProt features in nitrated proteins versus a non-nitrated protein background as determined using DAVID [41].

term	gene count	adjusted *p*-value
phosphoprotein	5325	1.9 × 10^–154^
coiled coil	1654	6.3 × 10^–95^
alternative splicing	5118	1.9 × 10^–54^
cytoplasm	2413	3.4 × 10^–46^
nucelotide binding	1299	6.9 × 10^–44^
ATP binding	1048	1.5 × 10^–43^
acetylation	1916	3.4 × 10^–37^
polymorphism	7508	2.8 × 10^–34^
nucleus	2955	6.7 × 10^–30^
cytoskeleton	507	9.2 × 10^–22^

To rule out any bias in gene functional enrichment, an equal number of genes were randomly selected for analysis in DAVID. As expected, no features were significantly enriched in these sets beyond *p* > 0.1 (data not shown), indicating that nitrated proteins are indeed enriched for specific functional classes.

### 4.4. Nitrated tyrosine residues are generally more solvent-accessible and more likely to occur in α-helices compared with non-nitratable tyrosine residues

**Figure 2.**
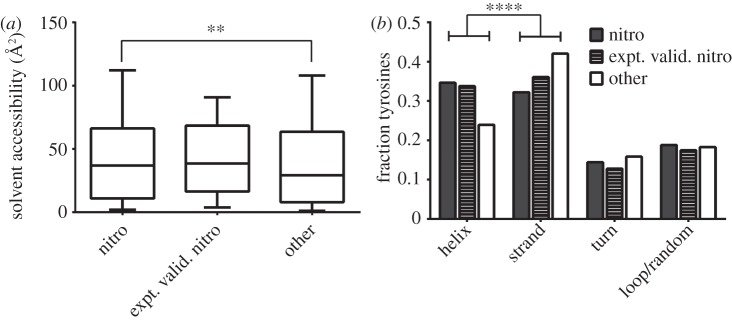
Structural analysis of nitrated tyrosine residues based on PDB X-ray crystal structures. (*a*) The solvent accessibility of predicted nitrated tyrosine residues (nitro) is significantly higher than all other tyrosine residues (other; **p = 0.0011, Mann–Whitney *U*-test). Experimentally validated nitrated tyrosine residues (expt. valid. nitro) similarly show an increase in solvent accessibility compared with other tyrosine residues, but the difference is not statistically significant (*p* = 0.0521, Mann–Whitney *U*-test). (*b*) Distribution of nitrated tyrosine and other tyrosine residues across the different protein secondary structure classes. The distribution of secondary structure between predicted nitrated tyrosine and other tyrosine is significantly different (figure 2*b*; *p* < 0.0001, *χ*^2^-test), with helices more enriched in nitrated tyrosine residues. Experimentally validated nitrated tyrosines again show a similar trend to the predicted tyrosine residues, but the difference is not significant compared with other tyrosine residues (*p* = 0.1979, *χ*^2^-test).

To determine the relationship between nitrated tyrosine the tertiary structure of proteins, all tyrosine residues were mapped to the PDB, where an X-ray crystal structure is available. Using DSSP, the solvent accessibility and secondary structure of each tyrosine was determined. For the analysis, tyrosine residues were separated into nitrated and non-nitrated. Comparing the distribution of solvent accessibility, predicted nitrated tyrosines are found to be significantly more solvent-accessible compared with non-nitrated tyrosine residues (figure 2*a*; *p* = 0.0011, Mann–Whitney *U*-test). Experiment validated nitrotyrosines showed a similar trend of higher solvent accessibility compared with non-nitrated tyrosine. However, the difference was not strongly significant (*p* = 0.0513, Mann–Whitney *U*-test). In terms of protein secondary structure, predicted nitrated tyrosine residues are distributed significantly differently to non-nitrated tyrosine residues (figure 2*b*; *p* < 0.0001, *χ*^2^-test). While experimentally validated nitrated tyrosines show a very similar trend, again the difference is not statistically significant (*p* = 0.1979, *χ*^2^-test). Specifically, predicted nitrated tyrosine residues are enriched in α-helices compared with β-strand secondary structures (*p* < 0.0001, *χ*^2^-test).

### 4.5. Helix propensity and charged amino acids are enriched adjacent to 3-nitrotyrosine residues

**Figure 3.**
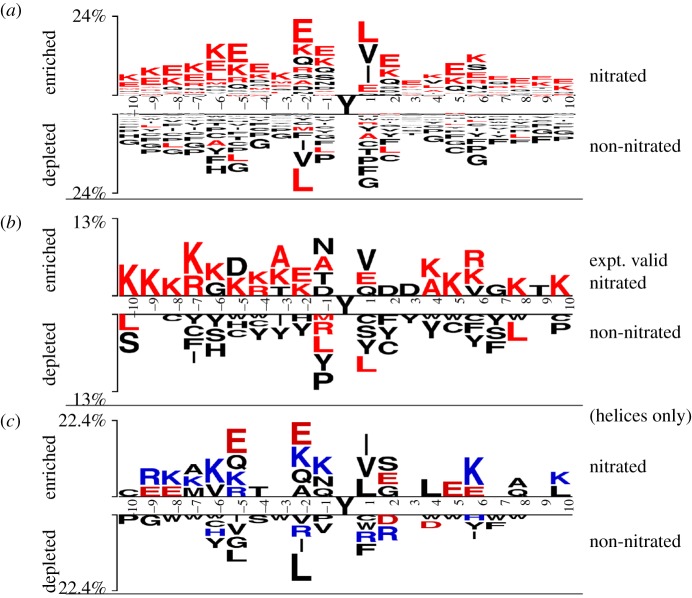
Two Sample Logos for 10 residues immediate adjacent to (*a*) predicted nitrated versus non-nitrated tyrosine residues and (*b*) experimentally validated nitrated tyrosine versus non-nitrated tyrosine residues. Amino acids with high propensity for occurrence in α-helices are coloured in red. (*c*) Two Sample Logos comparing predicted nitrated tyrosine and non-nitrated tyrosine residues that are located within helices. Positively charged residues are coloured blue while negatively charged residues are coloured red.

To determine whether there is indeed an enrichment of amino acids with propensity for α-helix formation adjacent to 3-nitrotyrosine residues, Two Sample Logos were created for ±10 residues adjacent to the tyrosine residues. Predicted or experimentally validated nitrated tyrosine residues were selected as the ‘positive sample’ and non-nitrated tyrosine as ‘negative sample’. The amino acids that have high propensity to be found in α-helices are shown in red in the logo. It is evident that high-helix-propensity amino acids are enriched surrounding both predicted and experimentally validated 3-nitrotyrosine compared with unmodified tyrosine residues (figure 3*a,b*). On the other hand, amino acids that have very low α-helix propensities, such as proline and glycine, are particularly depleted around predicted nitrated tyrosine residues (figure 3*a*). To investigate whether there are specific sequence features beyond the enrichment of high-helix-propensity amino acids around nitrated tyrosine residues, the sequence adjacent to the subset of nitrated and non-nitrated tyrosine residues that are present in helices were compared (figure 3*a*). Charged amino acids lysine and glutamic acid were most significantly enriched in the nitrated tyrosines located in helices.

### 4.6. Phosphotyrosine residues are more likely to be nitratable than by chance

The phosphorylation of tyrosine in proteins is one of the most important regulatory mechanisms in cell signalling. Many diseases (including cancer) arise through dysregulation of tyrosine kinases [49,50]. Nitration of tyrosine has been shown to abolish [22] or at least interfere with [21] the ability of tyrosine residues to be phosphorylated. It is therefore important to establish the susceptibility of phosphotyrosine residues to be nitrated in the human proteome.

**Figure 4.**
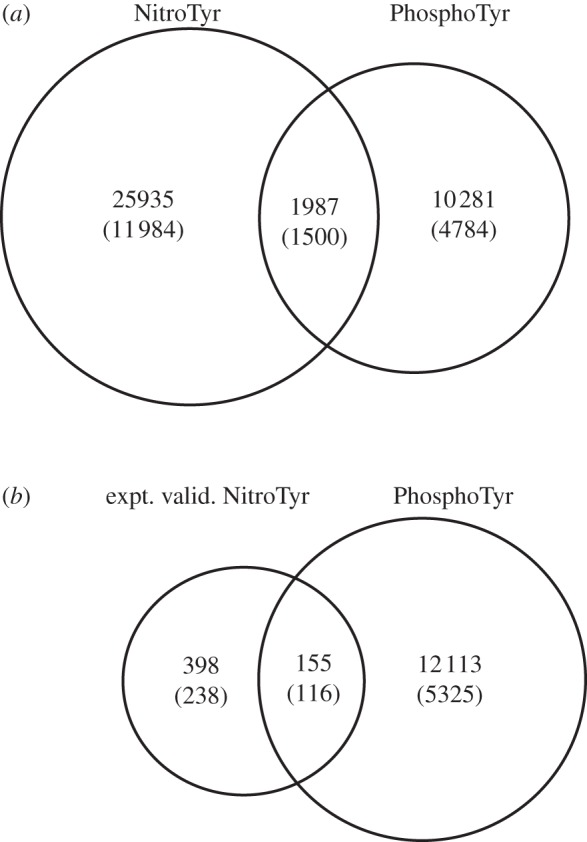
Venn diagram showing the overlap between (*a*) predicted nitrated tyrosine residues and phosphorylated tyrosine residues, and (*b*) experimentally validated nitrated tyrosine residues and phosphorylated tyrosine residues. The number of residues and proteins (in brackets) are shown. Bootstrapping of expected number of overlap between the phosphotryosine and nitrotyrosine show that the overlap is significantly higher than by chance alone (for both comparisons, *p* < 0.0001, one-sample *t*-test).

A total of 12 268 tyrosine residues from 5325 proteins were found to be annotated as phosphorylated in PhosphoSitePlus. Of these tyrosine residues, a total of 1987 (16.2%) were found to be predicted to be nitratable (figure 4*a*), while 155 (1.3%) have been shown to be nitrated experimentally (figure 4*b*). To determine whether this overlap is more statistically significant than can be expected by chance alone, 12 268 tyrosine residues were randomly selected from the total 301 091 tyrosine residues from the human proteome, and the overlap was determined with 3-nitrotyrosine residues. This bootstrapping process was repeated 500 times and resulted in an average overlap of 1280 ± 32 tyrosine residues between phosphorylated and predicted nitrated tyrosine, which is significantly less than the true overlap (*p* < 0.0001, one-sample *t*-test). The bootstrapped overlap between phosphorylated and experimentally validated nitrated tyrosine is 25 ± 3 tyrosine residues, which is also significantly less than the observed overlap (*p* < 0.0001, one-sample *t*-test).

### 4.7. Predicted nitrated tyrosines are less conserved than non-nitrated tyrosines while experimentally validated nitrated tyrosine residues are more conserved

**Figure 5.**
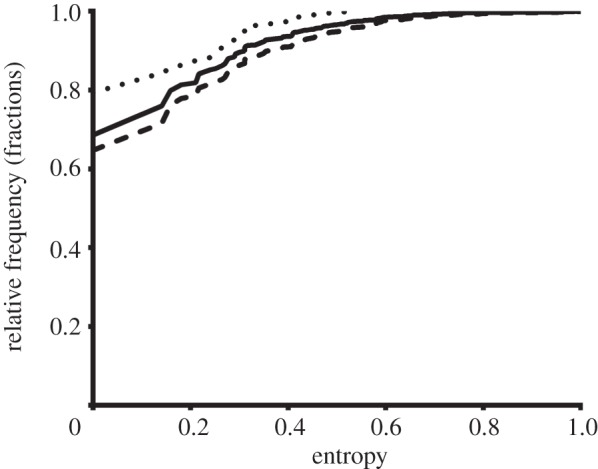
Cumulative frequency distribution of normalized Shannon's entropy of predicted nitrated tyrosine (dashed line), experimentally validated nitrated tyrosine (dotted line) and non-nitrated residues (solid line). Predicted nitrated tyrosine residues have significantly higher sequence entropy compared with non-nitrated tyrosine residues (*p* < 0.0001, Mann–Whitney *U*-test), as indicated by reaching higher entropy at lower cumulative frequency. Experimentally validated nitrated tyrosines have significantly lower entropy compared with non-nitrated tyrosine residues (*p* = 0.0026, Mann–Whitney *U*-test), as indicated by requiring the highest cumulative frequency to reach high entropy.

Examining amino acid conservation across mammals enables the conservation of tyrosine to be analysed over a reasonably large set of orthologous proteins. In total, the conservation of 91 342 tyrosine residues was examined. The tyrosine residues were annotated as nitrated, experimentally validated nitrated or non-nitrated. A summary of the conservation status of each type of tyrosine is shown in table 2. The level of conservation between predicted 3-nitrotyrosine and non-nitrated tyrosine residues was found to be significantly lower (*p* < 0.0001, *χ*^2^-test), while the conservation of experimentally validated 3-nitrotyrosine compared with non-nitrated tyrosine was significantly higher (*p* = 0.0028, *χ*^2^-test). To examine this trend further, the absolute entropy, *C*, across all sites was compared across the three tyrosine types (figure 5). Based on the cumulative frequency distribution, it is evident that predicted 3-nitrotyrosine residues show significantly higher sequence entropy compared with non-nitrated tyrosine residues (*p* < 0.0001, Mann–Whitney *U*-test), whereas experimentally validated 3-nitrotyrosine residues show significantly lower sequence entropy compared with non-nitrated tyrosine residues (*p* = 0.0026, Mann–Whitney *U*-test).

**Table 2.** Number and percentage of the nitrated and non-nitrated tyrosine residues that are conserved across orthologous proteins of 10 mammalian species. The conservation of predicted 3-nitrotyrosine (nitro) is significantly lower than non-nitrated tyrosine residues (non-nitrated) (*p* < 0.0001, *χ*^2^-test) while experimentally validated 3-nitrotyrosine (expt. valid. nitro) is significantly more conserved than non-nitrated residues (*p* = 0.0028, *χ*^2^-test).

	conserved	total
nitro	5586 (64.1%)	8718
expt. valid. nitro	141 (78.8%)	179
non-nitrated	56 205 (68.1%)	82 571

## 5. Discussion

While there are numerous studies that have demonstrated a role of the nitration of tyrosine in altering the function of specific proteins, whether this PTM plays a major role in normal physiological processes at a systems level has not been clear. The low abundance of 3-nitrotyrosine in the proteome has made the proteome-wide detection of the modification difficult, especially in *in vivo* systems. The lack of experimental data to date has made it unfeasible to perform a proteome-wide characterization of the biophysical and evolutionary properties of this PTM. Using GPS-YNO2, a recently developed algorithm that had been shown to be able to predict nitratable tyrosine with good accuracy, a set of putative nitratable tyrosine residues across the human proteome was generated. To further validate that the accuracy of GPS-YNO2 can be extrapolated to *in vivo* nitrated tyrosine sites, experimentally validated *in vivo* 3-nitrotyrosine residues were manually curated, and it was found that 85.7 per cent of all sites were also predicted by the algorithm. While it is difficult to determine the number of true negatives, it has been established by Liu *et al*. [40] that GPS-YNO2 has a specificity of 80.18 per cent. This suggested that predictions by GPS-YNO2 would generalize well for a proteome-wide analysis of biophysical and evolutionary trends of 3-nitrotyrosine residues.

By performing a comprehensive set of bioinformatics analyses on the set of nitratable tyrosine, the results show that there are some biophysical constraints on the prevalence of 3-nitrotyrosine residues. Specifically, nitrated tyrosine residues are generally more solvent-accessible than non-nitrated tyrosine and are more likely to be found in α-helices. Residues in β-strands are generally less solvent-accessible compared with α-helices [51] and thus the generally higher solvent accessibility in 3-nitrotyrosine compared with non-nitrated tyrosine is consistent with the enrichment of 3-nitrotyrosine found in helices. Since the nitration prediction algorithm used by GPS-YNO2 is dependent on biochemical/biophysical features of amino acids adjacent to tyrosine to predict nitration, the amino acid frequencies surrounding 3-nitrotyrosine were analysed, and it was found that amino acids that have the highest propensity to form α-helices, such as methionine, alanine, leucine, glutamate and lysine [44], are indeed generally enriched (figure 3*a,b*). Interestingly, when the sequence adjacent to nitrated and non-nitrated tyrosine residues that are found to be in helices are compared, further enrichment of charged amino acids, lysine and glutamic acid was found. While lysine and glutamic acid are already considered to be favourable for helix formation, their further enrichment around nitratable residues suggests that they may be important in promoting tyrosine nitration. Indeed, it has been suggested previously that negatively charged residues may play a role in determining the selectively of tyrosine nitration [37]. However, the role of positively charged lysine in tyrosine nitration is unclear.

Coiled coil is a tertiary structural motif in which a number of helices are coiled together in the form of typically a dimer or a trimer. Interestingly, performing biological feature enrichment analysis showed that coiled coils were enriched in nitrated proteins, a finding consistent with the enrichment of α-helices. Proteins with coiled coil motifs are important in gene regulation and are found in a number of oncogenic transcription factors, such as c-jun. The introduction of a bulky chemical group on tyrosine residues through nitration may affect DNA binding by affecting the affinity of the coiled coil on the negatively charged DNA, thereby altering transcriptional regulation.

Phosphotyrosine is a major functional PTM that has the potential to be significantly impaired by tyrosine nitration. Previous studies have shown that nitration of a tyrosine completely abolishes its ability to be phosphorylated [52]. This analysis shows that nitration is statistically more likely to affect phosphorylatable tyrosine residues than by chance alone. The increase in overlap between nitrated and phosphorlylated tyrosine is likely to be due to an enrichment of glutamic acid and leucine adjacent to many phosphotyrosine motifs [53], as these amino acids are also among the most frequently occurring adjacent 3-nitrotyrosine residues (figure 3). While there are few examples of direct interference of tyrosine phosphorylation by nitration *in vivo*, the results presented here suggest that 3-nitrotyrosine has an elevated potential to affect phosphotyrosine-mediated signalling pathways. It should be noted that many phosphotyrosine sites from PhosphoSitePlus [45] used in the current analysis are curated from high-throughput mass spectrometry-based studies where not all phosphotyrosines have been thoroughly validated. Therefore, the number of true sites overlapping with nitrotyrosine sites is likely to be lower than those observed in this study. Nevertheless, the overall trend of significantly increased overlap between phosphorylatable and nitratable tyrosine compared with chance alone, as observed in this study, is unlikely to change.

The results presented here demonstrate that there are biophysical characteristics that make 3-nitrotyrosine more likely to occur at specific locations within the proteome. On the other hand, the results also indicate that these biophysical characteristics are relatively mild. The number of nitratable tyrosine residues is correlated with the total tyrosine in proteins, suggesting that the biophysical constraints for tyrosine nitration are limited, and consequently it is unlikely that there are any specific processes that regulate tyrosine nitration at a proteome level. This is consistent with current research which has not yet identified specific enzymes involved in the nitration or the denitration of 3-nitrotyrosine. Furthermore, by analysing the conservation of tyrosine residues from human proteins with mammalian orthologues, it is evident that at the proteome level, predicted 3-nitrotyrosines are less conserved and have higher sequence entropy compared with non-nitrated tyrosine (table 2 and figure 5). This suggests that there is negative evolutionary selective pressure for nitratable tyrosine residues, and also suggests that, in the majority of cases, nitrotyrosine is probably not involved in normal cellular processes. This is consistent with the numerous studies that have implicated 3-nitrotyrosine in disease [9–14] and is further evidence that formation of 3-nitrotyrosine has a deleterious effect on cellular function. However, surprisingly, the subset of experimentally validated nitrated tyrosine shows a significantly higher level of conservation and has lower sequence entropy compared with non-nitrated tyrosine (table 2 and figure 5). One possible explanation for this observation is that mass spectrometry, the method used to experimentally identify nitrated tyrosine sites, preferentially detects highly expressed proteins [54], and it is known that highly expressed proteins generally have high sequence conservation [55]. Furthermore, the number of experimentally validated 3-nitrotyrosine sites with conservation data is low (total of 179 sites). This result nevertheless highlights that even small numbers of 3-nitrotyrosine have the potential to target the large number of highly conserved tyrosine residues. These conserved tyrosine residues are possibly functionally important and their nitration may underlie human diseases.

## 6. Conclusions

This study has demonstrated that there are biophysical constraints that influence the ability of a tyrosine to be nitrated, while, from an evolutionary perspective, this PTM probably has a deleterious effect on protein function. The biophysical constraints observed may arise due to specific chemistries that are favourable for the nitration of tyrosine, or perhaps the constraints dictate the physical accessibility of the tyrosine for nitration. In either case, the biophysical constraints appear to make 3-nitrotyrosine more likely to occur at α-helices and phosphorylatable tyrosine residues, meaning that spontaneous nitration as a result of oxidative/nitrosative stress does have an increased likelihood to interfere with gene regulation and signal transduction functions.

While this analysis has been performed using a set of predicted 3-nitrotyrosine sites in the human proteome, the fact that the prediction algorithm GPS-YNO2 achieved over 85.7 per cent accuracy in predicting endogenously nitrated tyrosine residues and overall 73.1 per cent of all experimentally determined nitrated tyrosine residues would suggest that the majority of the sites used in this proteome-wide analysis are likely to be reliable. Therefore, the findings described should reflect the actual occurrence of 3-nitrotyrosine in the human proteome.

## 7. Acknowledgements

This work is financially supported by an Australian Postgraduate Award (J.Y.N.), a Medical Advances Without Animals scholarship (J.Y.N.), a UNSW Vice-Chancellor's post-doctoral fellowship (J.W.H.W.) and a Cancer Institute NSW Early Career Fellowship (J.W.H.W.).

## Supplementary Material

Supplementary Table 1

## Supplementary Material

Supplementary Table 2

## Supplementary Material

Supplementary Table 3

## References

[1] Sokolovsky M Riordan JF Vallee BL 1966 Tetranitromethane: a reagent for the nitration of tyrosyl residues in proteins. Biochemistry 5, 3582–3589 10.1021/bi00875a029 (doi:10.1021/bi00875a029) 5339594

[2] Gaut JP Byun J Tran HD Heinecke JW 2002 Artifact-free quantification of free 3-chlorotyrosine, 3-bromotyrosine, and 3-nitrotyrosine in human plasma by electron capture-negative chemical ionization gas chromatography mass spectrometry and liquid chromatography-electrospray ionization tandem mass spectrometry. Anal. Biochem. 300, 252–259 10.1006/abio.2001.5469 (doi:10.1006/abio.2001.5469) 11779118

[3] Tohgi H Abe T Yamazaki K Murata T Ishizaki E Isobe C 1999 Alterations of 3-nitrotyrosine concentration in the cerebrospinal fluid during aging and in patients with Alzheimer's disease. Neurosci. Lett. 269, 52–54 10.1016/S0304-3940(99)00406-1 (doi:10.1016/S0304-3940(99)00406-1) 10821643

[4] Palazzolo-Ballance AM Suquet C Hurst JK 2007 Pathways for intracellular generation of oxidants and tyrosine nitration by a macrophage cell line. Biochemistry 46, 7536–7548 10.1021/bi700123s (doi:10.1021/bi700123s) 17530864 PMC2584613

[5] Ischiropoulos H Zhu L Beckman JS 1992 Peroxynitrite formation from macrophage-derived nitric oxide. Arch. Biochem. Biophys. 298, 446–451 10.1016/0003-9861(92)90433-W (doi:10.1016/0003-9861(92)90433-W) 1329657

[6] Huie RE Padmaja S 1993 The reaction of NO with superoxide. Free Radic. Res. Commun. 18, 195–199 10.3109/10715769309145868 (doi:10.3109/10715769309145868) 8396550

[7] Mohiuddin I Chai H Lin PH Lumsden AB Yao Q Chen C 2006 Nitrotyrosine and chlorotyrosine: clinical significance and biological functions in the vascular system. J. Surg. Res. 133, 143–149 10.1016/j.jss.2005.10.008 (doi:10.1016/j.jss.2005.10.008) 16360172

[8] Oury TD Tatro L Ghio AJ Piantadosi CA 1995 Nitration of tyrosine by hydrogen peroxide and nitrite. Free Radic. Res. 23, 537–547 10.3109/10715769509065275 (doi:10.3109/10715769509065275) 8574348

[9] Maciag AE Chakrapani H Saavedra JE Morris NL Holland RJ Kosak KM Shami PJ Anderson LM Keefer LK 2011 The nitric oxide prodrug JS-K is effective against non-small-cell lung cancer cells *in vitro* and *in vivo*: involvement of reactive oxygen species. J. Pharmacol. Exp. Ther. 336, 313–320 10.1124/jpet.110.174904 (doi:10.1124/jpet.110.174904) 20962031 PMC3033717

[10] Aslan M Dogan S 2011 Proteomic detection of nitroproteins as potential biomarkers for cardiovascular disease. J. Proteomics 74, 2274–2288 10.1016/j.jprot.2011.05.029 (doi:10.1016/j.jprot.2011.05.029) 21640858

[11] Lawless MW O'Byrne KJ Gray SG 2009 Oxidative stress induced lung cancer and COPD: opportunities for epigenetic therapy. J. Cell Mol. Med. 13, 2800–2821 10.1111/j.1582-4934.2009.00845.x (doi:10.1111/j.1582-4934.2009.00845.x) 19602054 PMC4498937

[12] Amann A Corradi M Mazzone P Mutti A 2011 Lung cancer biomarkers in exhaled breath. Expert Rev. Mol. Diagn. 11, 207–217 10.1586/erm.10.112 (doi:10.1586/erm.10.112) 21405971

[13] Sugiura H Komaki Y Koarai A Ichinose M 2008 Nitrative stress in refractory asthma. J. Allergy Clin. Immunol. 121, 355–360 10.1016/j.jaci.2007.11.009 (doi:10.1016/j.jaci.2007.11.009) 18158173

[14] Butterfield DA Reed T Sultana R 2011 Roles of 3-nitrotyrosine- and 4-hydroxynonenal-modified brain proteins in the progression and pathogenesis of Alzheimer's disease. Free Radic. Res. 45, 59–72 10.3109/10715762.2010.520014 (doi:10.3109/10715762.2010.520014) 20942567

[15] Yamakura F Taka H Fujimura T Murayama K 1998 Inactivation of human manganese-superoxide dismutase by peroxynitrite is caused by exclusive nitration of tyrosine 34 to 3-nitrotyrosine. J. Biol. Chem. 273, 14 085–14 089 10.1074/jbc.273.23.14085 (doi:10.1074/jbc.273.23.14085) 9603906

[16] Moreno DM Marti MA De Biase PM Estrin DA Demicheli V Radi R Boechi L 2011 Exploring the molecular basis of human manganese superoxide dismutase inactivation mediated by tyrosine 34 nitration. Arch. Biochem. Biophys. 507, 304–309 10.1016/j.abb.2010.12.011 (doi:10.1016/j.abb.2010.12.011) 21167124

[17] Vadseth C 2004 Pro-thrombotic state induced by post-translational modification of fibrinogen by reactive nitrogen species. J. Biol. Chem. 279, 8820–8826 10.1074/jbc.M306101200 (doi:10.1074/jbc.M306101200) 14681238

[18] Quint P Reutzel R Mikulski R McKenna R Silverman DN 2006 Crystal structure of nitrated human manganese superoxide dismutase: mechanism of inactivation. Free Radic. Biol. Med. 40, 453–458 10.1016/j.freeradbiomed.2005.08.045 (doi:10.1016/j.freeradbiomed.2005.08.045) 16443160

[19] Tao L 2006 Nitrative inactivation of thioredoxin-1 and its role in postischemic myocardial apoptosis. Circulation 114, 1395–1402 10.1161/CIRCULATIONAHA.106.625061 (doi:10.1161/CIRCULATIONAHA.106.625061) 16966583

[20] Yin T Hou R Liu S Lau WB Wang H Tao L 2010 Nitrative inactivation of thioredoxin-1 increases vulnerability of diabetic hearts to ischemia/reperfusion injury. J. Mol. Cell Cardiol. 49, 354–361 10.1016/j.yjmcc.2010.05.002 (doi:10.1016/j.yjmcc.2010.05.002) 20497906

[21] Gow AJ Duran D Malcolm S Ischiropoulos H 1996 Effects of peroxynitrite-induced protein modifications on tyrosine phosphorylation and degradation. FEBS Lett. 385, 63–66 10.1016/0014-5793(96)00347-X (doi:10.1016/0014-5793(96)00347-X) 8641468

[22] Kong SK Yim MB Stadtman ER Chock PB 1996 Peroxynitrite disables the tyrosine phosphorylation regulatory mechanism: lymphocyte-specific tyrosine kinase fails to phosphorylate nitrated cdc2(6–20)NH2 peptide. Proc. Natl Acad. Sci. USA 93, 3377–3382 10.1073/pnas.93.8.3377 (doi:10.1073/pnas.93.8.3377) 8622943 PMC39616

[23] Monteiro HP Arai RJ Travassos LR 2008 Protein tyrosine phosphorylation and protein tyrosine nitration in redox signaling. Antioxid. Redox Signal 10, 843–889 10.1089/ars.2007.1853 (doi:10.1089/ars.2007.1853) 18220476

[24] Mallozzi C Ceccarini M Camerini S Macchia G Crescenzi M Petrucci TC Di Stasi AM 2009 Peroxynitrite induces tyrosine residue modifications in synaptophysin C-terminal domain, affecting its interaction with *src*. J. Neurochem. 111, 859–869 10.1111/j.1471-4159.2009.06378.x (doi:10.1111/j.1471-4159.2009.06378.x) 19737347

[25] Brito C Naviliat M Tiscornia AC Vuillier F Gualco G Dighiero G Radi R Cayota AM 1999 Peroxynitrite inhibits T lymphocyte activation and proliferation by promoting impairment of tyrosine phosphorylation and peroxynitrite-driven apoptotic death. J. Immunol. 162, 3356–3366 10092790

[26] MacMillan-Crow LA Greendorfer JS Vickers SM Thompson JA 2000 Tyrosine nitration of c-SRC tyrosine kinase in human pancreatic ductal adenocarcinoma. Arch. Biochem. Biophys. 377, 350–356 10.1006/abbi.2000.1799 (doi:10.1006/abbi.2000.1799) 10845713

[27] Aburima A Riba R Naseem KM 2010 Peroxynitrite causes phosphorylation of vasodilator-stimulated phosphoprotein through a PKC dependent mechanism. Platelets 21, 421–428 10.3109/09537104.2010.483296 (doi:10.3109/09537104.2010.483296) 20624010

[28] Mondoro TH Shafer BC Vostal JG 1997 Peroxynitrite-induced tyrosine nitration and phosphorylation in human platelets. Free Radic. Biol. Med. 22, 1055–1063 10.1016/S0891-5849(96)00510-2 (doi:10.1016/S0891-5849(96)00510-2) 9034245

[29] Dekker F Abello N Wisastra R Bischoff R 2012 Enrichment and detection of tyrosine-nitrated proteins. Curr. Protoc. Protein Sci. 69, 14.13.1–14.13.19 10.1002/0471140864.ps1413s69 (doi:10.1002/0471140864.ps1413s69) 22851496

[30] MacMillan-Crow LA Thompson JA 1999 Immunoprecipitation of nitrotyrosine-containing proteins. Methods Enzymol. 301, 135–145 10.1016/S0076-6879(99)01076-9 (doi:10.1016/S0076-6879(99)01076-9) 9919561

[31] Zhan X Desiderio DM 2009 Mass spectrometric identification of *in vivo* nitrotyrosine sites in the human pituitary tumor proteome. Methods Mol. Biol. 566, 137–163 10.1007/978-1-59745-562-6_10 (doi:10.1007/978-1-59745-562-6_10) 20058170

[32] Tsikas D Mitschke A Gutzki FM 2012 Measurement of 3-nitro-tyrosine in human plasma and urine by gas chromatography-tandem mass spectrometry. Methods Mol. Biol. 828, 255–270 10.1007/978-1-61779-445-2_20 (doi:10.1007/978-1-61779-445-2_20) 22125150

[33] Tsikas D 2012 Analytical methods for 3-nitrotyrosine quantification in biological samples: the unique role of tandem mass spectrometry. Amino Acids 42, 45–63 10.1007/s00726-010-0604-5 (doi:10.1007/s00726-010-0604-5) 20495837

[34] Dremina ES Li X Galeva NA Sharov VS Stobaugh JF Schoneich C 2011 A methodology for simultaneous fluorogenic derivatization and boronate affinity enrichment of 3-nitrotyrosine-containing peptides. Anal. Biochem. 418, 184–196 10.1016/j.ab.2011.07.024 (doi:10.1016/j.ab.2011.07.024) 21855526 PMC3195362

[35] Zhang Q 2007 A method for selective enrichment and analysis of nitrotyrosine-containing peptides in complex proteome samples. J. Proteome Res. 6, 2257–2268 10.1021/pr0606934 (doi:10.1021/pr0606934) 17497906

[36] Ghesquiere B 2009 In vitro and in vivo protein-bound tyrosine nitration characterized by diagonal chromatography. Mol. Cell Proteomics 8, 2642–2652 10.1074/mcp.M900259-MCP200 (doi:10.1074/mcp.M900259-MCP200) 19741252 PMC2816017

[37] Souza JM Daikhin E Yudkoff M Raman CS Ischiropoulos H 1999 Factors determining the selectivity of protein tyrosine nitration. Arch. Biochem. Biophys. 371, 169–178 10.1006/abbi.1999.1480 (doi:10.1006/abbi.1999.1480) 10545203

[38] Abello N Kerstjens HA Postma DS Bischoff R 2009 Protein tyrosine nitration: selectivity, physicochemical and biological consequences, denitration, and proteomics methods for the identification of tyrosine-nitrated proteins. J. Proteome Res. 8, 3222–3238 10.1021/pr900039c (doi:10.1021/pr900039c) 19415921

[39] Elfering SL Haynes VL Traaseth NJ Ettl A Giulivi C 2004 Aspects, mechanism, and biological relevance of mitochondrial protein nitration sustained by mitochondrial nitric oxide synthase. Am. J. Physiol. Heart Circ. Physiol. 286, H22–H29 10.1152/ajpheart.00766.2003 (doi:10.1152/ajpheart.00766.2003) 14527943

[40] Liu Z Cao J Ma Q Gao X Ren J Xue Y 2011 GPS-YNO2: computational prediction of tyrosine nitration sites in proteins. Mol. Biosyst. 7, 1197–1204 10.1039/c0mb00279h (doi:10.1039/c0mb00279h) 21258675

[41] Huang DW Sherman BT Lempicki RA 2009 Systematic and integrative analysis of large gene lists using DAVID bioinformatics resources. Nat. Protoc. 4, 44–57 10.1038/nprot.2008.211 (doi:10.1038/nprot.2008.211) 19131956

[42] Kabsch W Sander C 1983 Dictionary of protein secondary structure: pattern recognition of hydrogen-bonded and geometrical features. Biopolymers 22, 2577–2637 10.1002/bip.360221211 (doi:10.1002/bip.360221211) 6667333

[43] Vacic V Iakoucheva LM Radivojac P 2006 Two Sample Logo: a graphical representation of the differences between two sets of sequence alignments. Bioinformatics 22, 1536–1537 10.1093/bioinformatics/btl151 (doi:10.1093/bioinformatics/btl151) 16632492

[44] Pace CN Scholtz JM 1998 A helix propensity scale based on experimental studies of peptides and proteins. Biophys. J. 75, 422–427 10.1016/S0006-3495(98)77529-0 (doi:10.1016/S0006-3495(98)77529-0) 9649402 PMC1299714

[45] Hornbeck PV Chabra I Kornhauser JM Skrzypek E Zhang B 2004 PhosphoSite: a bioinformatics resource dedicated to physiological protein phosphorylation. Proteomics 4, 1551–1561 10.1002/pmic.200300772 (doi:10.1002/pmic.200300772) 15174125

[46] Waterhouse RM Zdobnov EM Tegenfeldt F Li J Kriventseva EV 2011 OrthoDB: the hierarchical catalog of eukaryotic orthologs in 2011. Nucleic Acids Res. 39, D283–D288 10.1093/nar/gkq930 (doi:10.1093/nar/gkq930) 20972218 PMC3013786

[47] Thompson JD Higgins DG Gibson TJ 1994 CLUSTAL W: improving the sensitivity of progressive multiple sequence alignment through sequence weighting, position-specific gap penalties and weight matrix choice. Nucleic Acids Res. 22, 4673–4680 10.1093/nar/22.22.4673 (doi:10.1093/nar/22.22.4673) 7984417 PMC308517

[48] Johansson F Toh H 2010 A comparative study of conservation and variation scores. BMC Bioinform. 11, 388 10.1186/1471-2105-11-388 (doi:10.1186/1471-2105-11-388) PMC2920274 20663120

[49] Sun T 2011 Activation of multiple proto-oncogenic tyrosine kinases in breast cancer via loss of the PTPN12 phosphatase. Cell 144, 703–718 10.1016/j.cell.2011.02.003 (doi:10.1016/j.cell.2011.02.003) 21376233 PMC6014607

[50] Linger RM Keating AK Earp HS Graham DK 2008 TAM receptor tyrosine kinases: biologic functions, signaling, and potential therapeutic targeting in human cancer. Adv. Cancer Res. 100, 35–83 10.1016/S0065-230X(08)00002-X (doi:10.1016/S0065-230X(08)00002-X) 18620092 PMC3133732

[51] Lins L Thomas A Brasseur R 2003 Analysis of accessible surface of residues in proteins. Protein Sci. 12, 1406–1417 10.1110/ps.0304803 (doi:10.1110/ps.0304803) 12824487 PMC2323943

[52] Takakusa H Mohar I Kavanagh TJ Kelly EJ Kaspera R Nelson SD 2012 Protein tyrosine nitration of mitochondrial carbamoyl phosphate synthetase 1 and its functional consequences. Biochem. Biophys. Res. Commun. 420, 54–60 10.1016/j.bbrc.2012.02.114 (doi:10.1016/j.bbrc.2012.02.114) 22402285 PMC3359619

[53] Amanchy R Periaswamy B Mathivanan S Reddy R Tattikota SG Pandey A 2007 A curated compendium of phosphorylation motifs. Nat. Biotechnol. 25, 285–286 10.1038/nbt0307-285 (doi:10.1038/nbt0307-285) 17344875

[54] Wu L Han DK 2006 Overcoming the dynamic range problem in mass spectrometry-based shotgun proteomics. Expert Rev. Proteomics 3, 611–619 10.1586/14789450.3.6.611 (doi:10.1586/14789450.3.6.611) 17181475

[55] Drummond DA Bloom JD Adami C Wilke CO Arnold FH 2005 Why highly expressed proteins evolve slowly. Proc. Natl Acad. Sci. USA 102, 14 338–14 343 10.1073/pnas.0504070102 (doi:10.1073/pnas.0504070102) PMC1242296 16176987

